# Diabetes mellitus risk prediction in the presence of class imbalance using flexible machine learning methods

**DOI:** 10.1186/s12911-022-01775-z

**Published:** 2022-02-10

**Authors:** Somayeh Sadeghi, Davood Khalili, Azra Ramezankhani, Mohammad Ali Mansournia, Mahboubeh Parsaeian

**Affiliations:** 1 Department of Epidemiology and Biostatistics, School of Public Health, Tehran University of Medical Sciences, P.O. Box 14155-6446, Tehran, Iran; 2 Prevention of Metabolic Disorders Research Center, Research Institute for Endocrine Sciences, Shahid Beheshti University of Medical Sciences, Tehran, Iran; 3 Department of Biostatistics and Epidemiology, Research Institute for Endocrine Sciences, Shahid Beheshti University of Medical Sciences, Tehran, Iran

**Keywords:** Diabetes mellitus, Machine learning, Imbalanced data, Sampling strategies, Cost-sensitive learning

## Abstract

**Background:**

Early detection and prediction of type two diabetes mellitus incidence by baseline measurements could reduce associated complications in the future. The low incidence rate of diabetes in comparison with non-diabetes makes accurate prediction of minority diabetes class more challenging.

**Methods:**

Deep neural network (DNN), extremely gradient boosting (XGBoost), and random forest (RF) performance is compared in predicting minority diabetes class in Tehran Lipid and Glucose Study (TLGS) cohort data. The impact of changing threshold, cost-sensitive learning, over and under-sampling strategies as solutions to class imbalance have been compared in improving algorithms performance.

**Results:**

DNN with the highest accuracy in predicting diabetes, 54.8%, outperformed XGBoost and RF in terms of AUROC, g-mean, and f1-measure in original imbalanced data. Changing threshold based on the maximum of f1-measure improved performance in g-mean, and f1-measure in three algorithms. Repeated edited nearest neighbors (RENN) under-sampling in DNN and cost-sensitive learning in tree-based algorithms were the best solutions to tackle the imbalance issue. RENN increased ROC and Precision-Recall AUCs, g-mean and f1-measure from 0.857, 0.603, 0.713, 0.575 to 0.862, 0.608, 0.773, 0.583, respectively in DNN. Weighing improved g-mean and f1-measure from 0.667, 0.554 to 0.776, 0.588 in XGBoost, and from 0.659, 0.543 to 0.775, 0.566 in RF, respectively. Also, ROC and Precision-Recall AUCs in RF increased from 0.840, 0.578 to 0.846, 0.591, respectively.

**Conclusion:**

G-mean experienced the most increase by all imbalance solutions. Weighing and changing threshold as efficient strategies, in comparison with resampling methods are faster solutions to handle class imbalance. Among sampling strategies, under-sampling methods had better performance than others.

## Introduction

Diabetes mellitus (DM) is a chronic disease and according to the International Diabetes Federation (IDF), it is one of the fastest growing global health emergencies in this century. About 463 million diabetic people lived worldwide in 2019, of whom 352 million people are of working age (between 20 and 64 years old). It is projected 417 million adults will live with diabetes by 2030. In 2019, the proportion of undiagnosed diabetes is estimated at 50.1% around the world. Untreated diabetes can damage the heart, kidneys, nerves and can cause eye difficulties such as diabetic retinopathy [1]. According to IDF, in 2019, total health expenditures for diabetes was 760.3 billion dollars and it is expected to increase to 824.7 billion dollars by 2030 [2]. Identifying at-risk people, in addition to prevent health problems and promote quality of life, can save billions of dollars.

In recent years, machine learning methods, specifically deep neural networks have provided considerable applications in the health system [3–7]. Machine learning algorithms can model complicated and nonlinear patterns to identify at-risk people. In addition, some algorithms could extract and determine features importance [8, 9]. Healthcare researchers are often interested in predicting disease cases which are rare in comparison with normal population. As a result, class imbalance is a common issue in most medical datasets. In the presence of class imbalance, minority class has a lower significant number of instances relative to other class. Most classifiers aim to achieve optimal performance on the whole classes. It has been proved that algorithms tend not to perform well on the minority class [10, 11]. There are several reasons for the poor results of learning algorithms in the classification of minority class. Rare samples may be treated as noisy, small sample size could cause challenges for models to detect rare patterns and evaluation metrics are biased towards the majority class [12, 13]. In the healthcare applications, misclassification minority class of patients, impose more costs than an error in classifying healthy persons. However, standard learning algorithms mostly assume an equal misclassification error and balanced class distribution [14]. Analyzing diabetic data to predict occurrence of diabetes mostly has been challenging. Complex and non-linear patterns of risk factors, in addition to the imbalance distribution of diabetes, are big issues in the prediction models.

To cope with the class imbalance problem, two main approaches have been established in the literature [15]. At data level, class distribution of data becomes fairly balanced with sampling techniques [16, 17]. At algorithm level, the distribution of data remains unchanged, but by modifying the cost of misclassification in minority class, model has been adjusted to focus more on learning rare class [18]. In threshold moving which is categorized under the algorithm level approach, class label prediction will be based on the optimal threshold instead of the default threshold (0.5) which is used routinely [15].

In this study, we will evaluate three the state-of-the-art machine learning algorithms, deep neural network (DNN), extreme gradient boosting (XGBoost), and random forest with various imbalance solving strategies including sampling methods, cost-sensitive learning, and threshold moving to improve prediction accuracy for the risk of diabetes. We will compare the effect of each strategy on algorithms performance based on various metrics and determine the best solution.

## Methods and materials

### Data description

We used data from the Tehran Lipid and Glucose Study (TLGS) which its details have been published previously [19–21]. Briefly, this study aims to determine atherosclerosis risk factors on a representative sample of district-13 of Tehran residents (n = 15,005, age $\ge$ 3) that started at 1999–2001 as cross-sectional prevalence study (phase 1). To determine the efficacy of population-based measures in preventing the incidence of diabetes mellitus and dyslipidemia, lifestyle intervention implemented in selected people that started at 2002–2005 as prospective follow-up study (phase 2). Data of all participants measured repeatedly every three years. The TLGS study was approved by the ethics committee at the Research Institute for Endocrine Sciences at Shahid Beheshti University of Medical Sciences. The study procedure and its aims were explained to all participants prior to data collection, and all participants in the study provided informed consent. All methods were carried out in accordance with relevant guidelines and regulations. Approval for undertaking the current project was also obtained from the Research Institute for Endocrine Sciences, Shahid Beheshti University of Medical Sciences. Non-diabetic people who aged > 20 years were selected from phase 3 (2005–2008) (i.e. second re-examination) of this population-based ongoing study. These subjects were followed for the next three phases (phase 4, 2008–2011, phase 5, 2011–2014, phase 6, 2014–2017). During phases 4 to 6, 315, 326, and 326 new cases of DM type-2 were identified respectively. Type-2 diabetes was defined based on fasting plasma glucose (FPG) $\ge$ 126 mg/dL or 2 h post-challenge plasma glucose (2 h-PG) $\ge$ 200 mg/dL or taking antidiabetic drugs. We considered people diabetic if they had one of the mentioned conditions in any 3 consecutive phases. Nondiabetic subjects who were lost to follow-up in the last phase were discarded because we could not consider them surely nondiabetic by the end of follow-up. Hence 1930 individuals were eliminated from 7600. In the end, 967 of the 5670 subjects were diagnosed with type-2 diabetes, while 4703 of the subjects were nondiabetic.

All selected variables for the study included demographic, anthropometric measures, physical activity, family history of CVD and Diabetes, biochemical blood parameters, systolic and diastolic blood pressure, smoking status, and medication for hypertension and hyperlipidemia. The dependent variable was the incidence of diabetes during the 9-year follow-up period.

### Data preprocessing

To detect outliers in this high dimensional data we used the Isolation Forest method. It is an efficient way in identifying outliers based on random forest. When a sample is an outlier, it will be isolated in a shorter path than a normal sample in recursive splitting in the fitted decision trees [22].

To impute missing values, we implemented a multivariate iterative method based on extremely randomized tree (extra tree) regressors. In this method, each feature with missing values is considered as a dependent variable and other features are predictors in a regression model. It repeated iteratively for each feature and for a certain number of rounds. We used extra tree classifier for categorical features, and extra tree regressors for continuous variables. Extra tree algorithm is an ensemble of randomized decision trees on various sub-samples of the dataset [23, 24].

Since the distribution of classes is imbalanced, stratified split with 70% for training and 30% for testing is used. With stratified split strategy, the ratio of diabetics to nondiabetics individuals remains the same in train and test data. All preprocessing methods included outlier detection and imputing parameters are only learned from training data and then transformed to testing data. It prevents information leakage from testing data to the learning process that could lead to an optimistic evaluation of models performance. It means that testing dataset had no contribution to the learning process and only have used for evaluating final models performance.

All programming was carried out in Python version 3.6 using Scikitlearn, Imbalanced-learn, Keras, and other related libraries.

### Machine learning algorithms

To compare various algorithms performance in predicting the patients with diabetes, we applied deep neural network, extremely gradient boosting, and random forest methods.

#### Algorithms

***Deep neural network*** In neural network independent variables input to the first layer, all neurons in this layer are fully connected to neurons in subsequent layers which are called hidden layers. The last layer outputs the prediction of the network. Deep neural networks have more than one hidden layer. Each neuron is weighted, and a bias value is added to the summation of weighted neurons. Weights control the contribution of each neuron in learning the network. In a neural network architecture, first, initial random weights are assigned to input neurons, then an activation function is used to calculate the output of each neuron in the hidden layer.

1 $$\mathrm{f}\left(\mathrm{x},\mathrm{w}\right)=\upphi \left(\sum_{j=1}^{n}{w}_{ij}{x}_{j}+{b}_{i}\right)$$

in this formula, x represents the value of neuron in the layer, and w represents the corresponding weight which after their multiplication, the sum of them is added to a bias term. Then activation function is applied to this value. The most popular activation function in hidden layers is Rectified Linear Unit (ReLU), which is calculated as follows:

2 $$\phi \left(x\right)=\mathrm{max}\left(0,x\right)$$

**Fig. 1:**
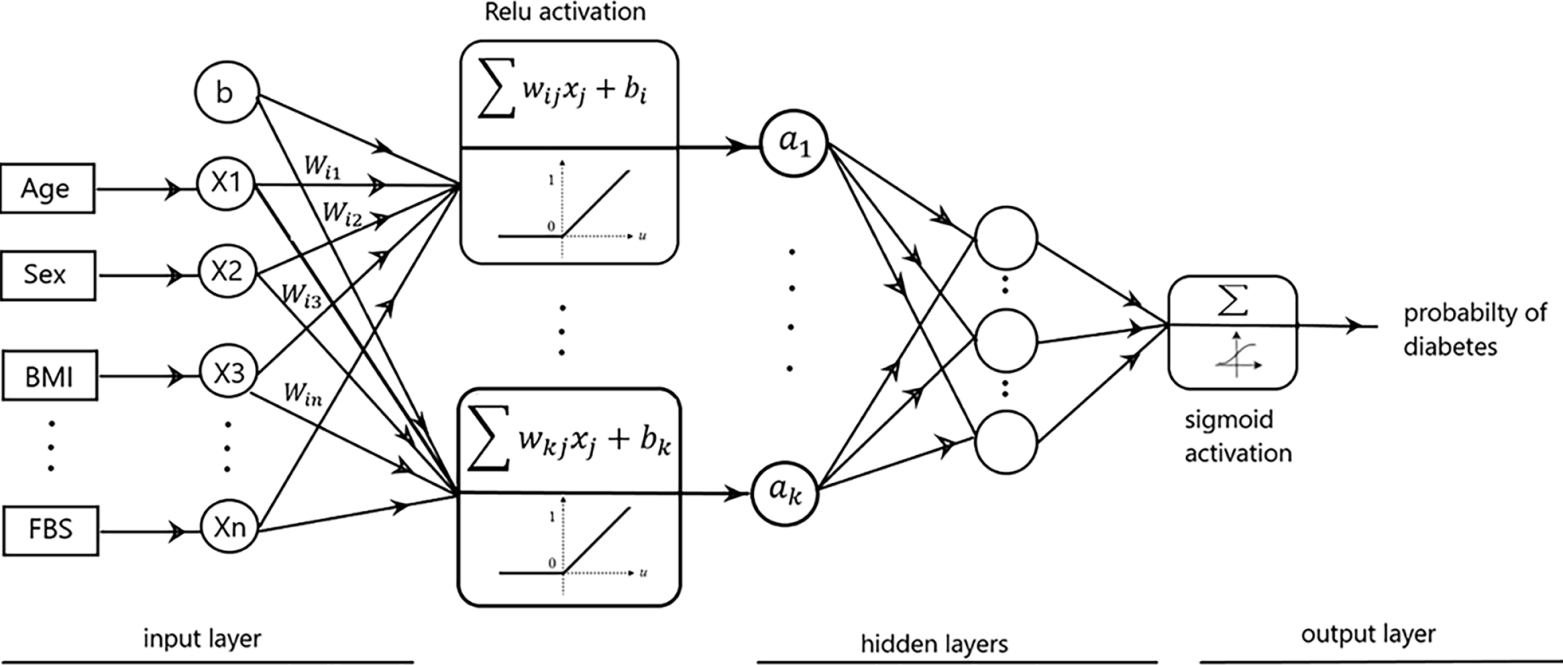
DNN structure for classification diabetes

ReLU solves the traditional vanishing gradient problem in learning deep neural networks [25]. For non-negative values, it simply returns the input value. Because output of each layer is the input of the next layer, applying multiple activation functions connected in a chain, represent nonlinear and complex relations between variables. In the last layer, sigmoid activation function is applied to project values to the range from 0 to 1 (Fig. 1). This estimated value illustrates the probability of being diabetic for the input variables.

Concerning the error of the network in predicting of being diabetic for each individual, initial weights will be updated to reach stop criteria. To prevent overfitting and to increase generalization of the trained model to unseen data, we used early stopping and drop out in learning of the model. By increasing the number of neurons in hidden layers, training error decreases, but testing error after some point increases. Drop out is a type of regularization which randomly deactivate a fraction of neurons and all connections of them in a hidden layer during the learning process. With this approach, in each iteration of training, some neurons are omitted from learning, so, different neurons contribute to train model and it leads to an ensemble of sub-networks. Each sub-network could learn a different aspect of data. Early stopping is another kind of regularization and it stops the learning process when performance of the model starts to decrease in hold out validation data [26].

**Fig. 2:**
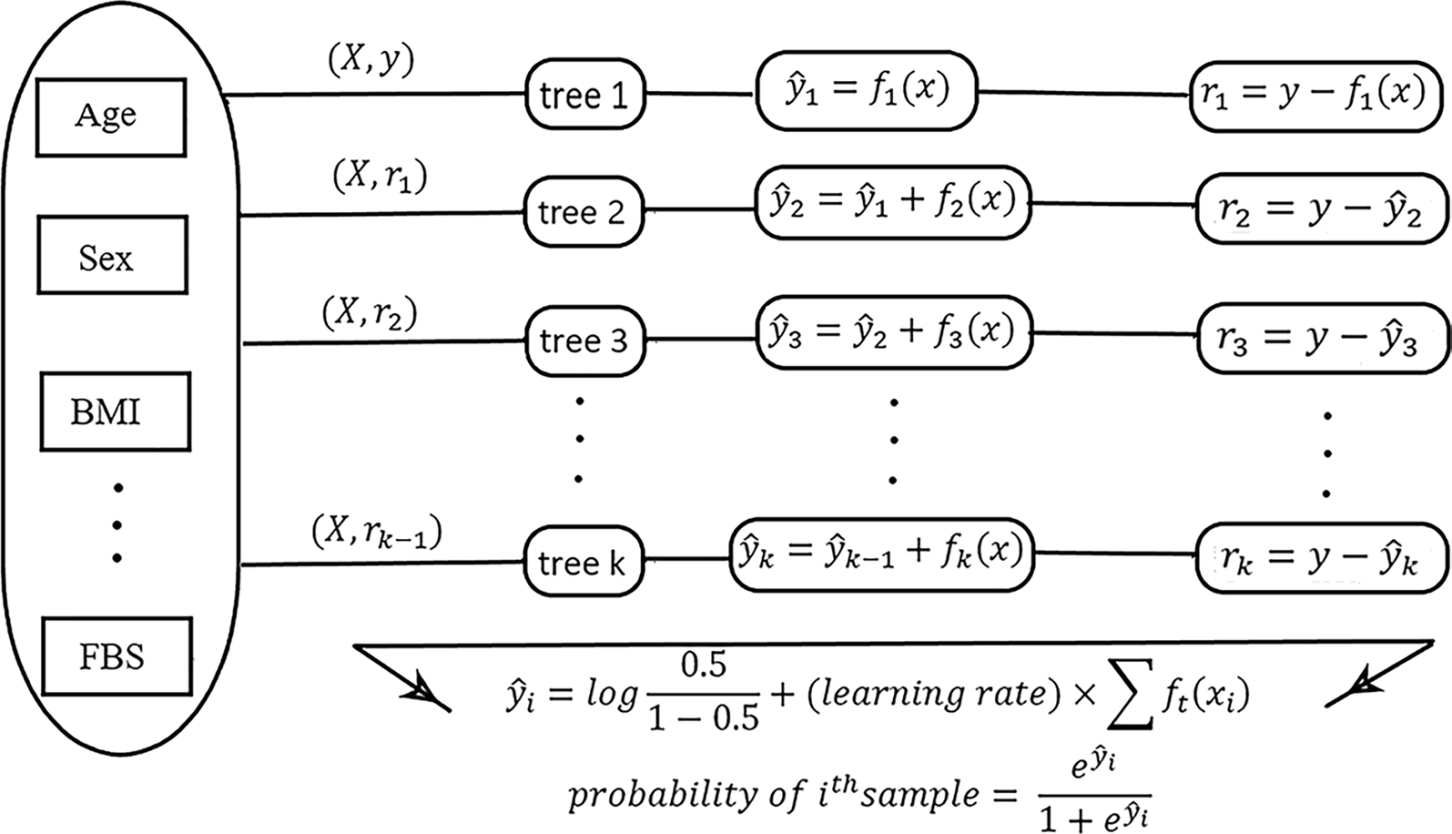
XGBoost structure for classification diabetes

*Extreme gradient boosting* It is an efficient implementation of gradient boosting algorithm. Classification and regression trees (CART), which assign a prediction score to each leaf, are the base learners. First, an initial value is assumed as prediction. This prediction is improved by adding a new tree to the residuals of its predecessor tree. This structure is showed in Fig. 2. After learning each tree, its contribution to the final model is weighted by the learning rate which is commonly between 0.1 and 0.3. In addition to the use of regularization term and shrinking learning rate to reduce overfitting, in XGBoost we can implement sub-sample of columns and rows before creating each tree [27].

*Random forest* It is an ensemble of decision trees which are constructed based on bootstrap samples. Each tree is learned by a random sample that is taken with replacement from training data. In the presence of a strong predictor, most of constructed trees use this predictor in the top split [28]. In random forest algorithm each split is built based on a random subsample of predictors. By this approach, all predictors take chance in learning data and model generalization to unseen data is increased. In classification, the most predicted class is the final prediction of the ensemble model.

#### Evaluation metrics

Accuracy measures overall performance of the algorithm, but in imbalanced data, this metric can be misleading. If algorithm always classify all samples as majority class, accuracy will be as high as the ratio of majority class, but definitely, this algorithm is useless. G-mean is geometric mean of sensitivity and specificity. Poor performance in diabetic class leads to a low g-mean, even if all non-diabetic persons correctly be predicted. F1-measure is a harmonic mean of recall (sensitivity) and precision that weighs precision and recall equally. Matthews Correlation Coefficient (MCC) is robust to data imbalance. It is a discretization of Pearson correlation between the observed and predicted classes [29]. Receiver Operating Characteristic (ROC) curve represents sensitivity (recall) versus 1-specificity for all possible thresholds. Area under it (AUC) is summary of this curve [30]. Precision-Recall (P-R) curve represents precision versus recall for all possible thresholds. In imbalanced datasets, P-R curve is more informatics than ROC curve [31]. In the case of focusing on classification successes, g-mean is not biased towards the majority class. But, if we also want to consider classification errors, MCC is preferred [29]. Selecting a suitable metric to determine best algorithm, always have been challenging [32].

#### Parameter and feature selection

To determine hyper-parameters (these parameters are specified by the analyst in order to optimize the performance of the model, and they cannot be estimated from the data) of classifiers, we used fivefold stratified cross-validation grid search. In this method, all possible values of different parameters are considered. Then, for each combination of these values, the model is fitted to four training folds and evaluated by a remained test fold. Finally, the average of the results is considered. The combination which leads to highest g-mean is chosen as the best hyper-parameters.

After selecting optimal values of hyper-parameters, to determine the most important features, we used SHAP (SHapley Additive exPlanation) [33] values which could explain black-box machine learning algorithms. Shapley value as a concept in the game theory, calculates each player contribution to the final team result. It is the average marginal contribution of each player by considering all possible combination of players. For machine learning algorithms, SHAP estimates Shapley values to determine each features contribution to the output of the model.

#### Tackling class imbalance

*Threshold moving* This is the simplest way in handling class imbalance [34]. In unequal class distribution and costs of misclassification, default threshold (0.5) is not appropriate in the prediction of class labels. The optimized threshold can be selected based on max of g-mean in ROC or f1-measure in P-R curves. These two approaches yield different thresholds.

*Cost-sensitive learning* Learning of algorithms are based on minimizing loss function. Each instance of the training dataset has equal weight in updating unknown parameter values during the iterative learning process of the algorithm. by assigning higher weights to minority class, and minimizing weighted loss function, instances from this class will have a greater role in the learning process [35].

*Sampling* Repeated edited nearest neighbors (RENN) as an under-sampling method is a strategy to remove noisy, redundant, and borderline samples. Each instance in majority class is classified by its k nearest neighbors. If sample is misclassified by its neighbors, it will be removed, otherwise this sample is remained. In repeated edited nearest neighbors this editing is repeated several times [36].

One sided selection (OSS) as an under-sampling method selects all minority class samples, and one randomly chosen sample form majority class combined to construct a new, smaller training set (C). Then, all original training samples are classified by 1 nearest neighbor classifier. Each sample from majority class which is misclassified by its nearest neighbor will be added to C. In the next step, all majority Tomek links samples which are the nearest neighbors from different classes [34] are removed from C. As a result, the under-sampled training set (C) contains all minority class samples in addition to cleaned set of the majority class from redundant, noisy-borderline samples [37].

Synthetic minority oversampling technique (SMOTE) generates synthetic examples by operation in feature space [16]. For oversampling, an instance and its nearest neighbors are randomly selected. Then, based on the desired amount of oversampling, some neighbors are chosen at random. After that, the difference between the selected sample and its neighbor in the feature space is taken. This difference is multiplied by a random number from (0,1) distance and is added to the selected instance. By this approach, synthetic samples are generated between two neighbors in the minority class.

**Fig. 3:**
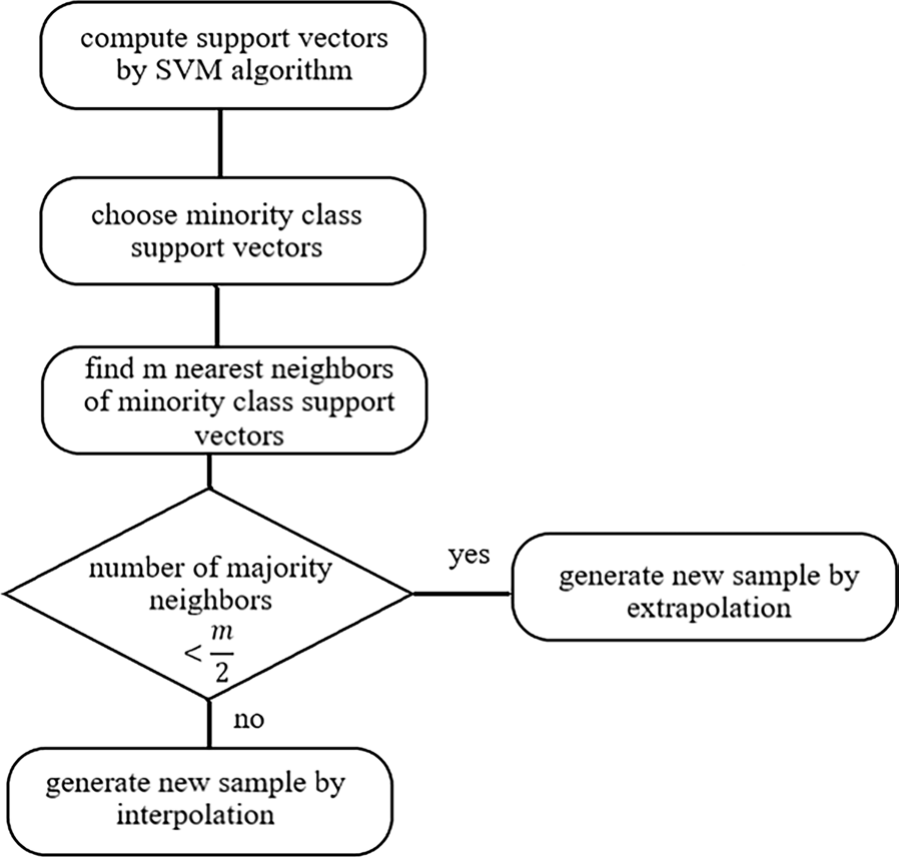
The flowchart of the SVM-SMOTE algorithm

*SVM-SMOTE* this method only oversample instances from minority class which are in borderline. To identify borderline instances, support vector machine (SVM) algorithm is applied. SVM finds the best hyperplane that separates samples of two classes with maximum margins. This optimal hyperplane is only found based on a few samples which are called support vectors. In SVM-SMOTE, samples from minority class that are around this borderline support vectors are oversampled by interpolation and extrapolation. In this algorithm, based on the number of nearest neighbors of majority class around minority class support vectors, oversampling is applied. If most of the m nearest neighbors of chosen minority support vector are from the majority class, as SMOTE strategy, new samples are generated by interpolation. But, if less than a half of m nearest neighbors are from the majority class, SMOTE oversampling is applied by extrapolation (Fig. 3) [38].

*Hybrid approach* class balance can be improved by combination of under and oversampling. ENN-SMOTE is a hybrid technique that performs under-sampling in majority class by edited nearest neighbor method and oversampling in minority class by SMOTE.

## Results

From 5670 samples considered in this study, 57 samples have identified as outliers and have been discarded. Family history of cardiovascular disease (CVD) and diabetes, and also being exposed to second-hand smoke at home or work had respectively 29, 24, and 18 percent of missing values. Other variables had less than 3 percent missing values. Diabetic class percentage was 16.5% in training data. Characteristics of individuals at baseline (phase 3) has been summarized in Table 1.

**Table 1:** Baseline characteristics of adult participants of the Tehran Lipid and Glucose Study in phase 3

Categorical variables	Diabetic individuals	Non-diabetic individuals	Total population
	Number (%)	Number (%)	Number (%)
**Sex**			
Women	522 (56.3)	2690 (57,4)	3212 (57.2)
Men	405 (43.7)	1996 (42.6)	2401 (42.8)
**Marital status**			
Single	47 (5.1)	788 (16.8)	835 (14.9)
Married	819 (88.3)	3707 (79.1)	4526 (80.7)
Divorced	8 (0.9)	60 (1.3)	68 (1.2)
Widowed	53 (5.7)	129 (2.8)	182 (3.2)
**Education**			
High (> 12 years)	135 (14.8)	1034 (22.4)	1169 (21.1)
Moderate (6–12 years)	478 (52.35)	2756 (59.7)	3234 (58.4)
Low (< 6 years)	300 (32.85)	830 (18)	1130 (20.4)
**Smoking status**			
Never or in the past	809 (88.8)	4137 (89.6)	4946 (89.5)
Current	102 (11.2)	480 (10.4)	582 (10.5)
**Exposed to second-hand smoke at home or work**			
No	688 (84.7)	3357 (81.1)	4045 (81.7)
Yes	124 (15.3)	783 (18.9)	907 (18.3)
**Physical activity**			
Low	331 (36.7)	1647 (36.1)	1978 (36.2)
High	570 (63.7)	2918 (63.9)	3488 (63.8)
**Family history of diabetes**			
No	351 (45.3)	2001 (57.4)	2352 (55.2)
Yes	424 (54.7)	1484 (42.6)	1908 (44.8)
**Use of lipid-lowering drug**			
No	884 (95.4)	4584 (97.8)	5648 (97.4)
Yes	43 (4.6)	102 (2.2)	145 (2.6)
**Use of antihypertensive drug**			
No	878 (94.7)	4595 (98.1)	5473 (97.5)
Yes	49 (5.3)	91 (1.9)	140 (2.5)
**Family history of CVD**			
No	457 (66.6)	2371 (72.4)	2828 (71.4)
Yes	229 (33.4)	902 (27.6)	1131 (28.6)

Continuous variables	Mean ± SD	Mean ± SD	Mean ± SD
Age (years)	49.56 ± 13.58	40.60 ± 12.89	42.09 ± 13.42
Height (cm)	161.35 ± 9.46	163.34 ± 9.53	163.01 ± 9.54
Weight (kg)	78.13 ± 13.75	71.55 ± 13.19	72.64 ± 13.51
Waist (cm)	97.71 ± 11.36	88.79 ± 12.22	90.27 ± 12.53
BMI (kg/m^2^)	30.04 ± 4.95	26.80 ± 4.37	27.33 ± 4.63
Waist-hip ratio	0.94 ± 0.083	0.88 ± 0.094	0.89 ± 0.094
Systolic blood pressure (mm HG)	120.90 ± 18.24	110.61 ± 15.26	112.31 ± 16.25
Diastolic blood pressure (mm HG)	77.10 ± 10.42	72.23 ± 9.94	73.04 ± 10.18
Fasting plasma glucose (mg/dL)	97.80 ± 10.61	87.10 ± 7.47	88.87 ± 8.99
Two-hour postprandial plasma glucose (mg/dL)	127.64 ± 33.18	96.42 ± 23.18	101.58 ± 27.65
High-density lipoprotein (mg/dL)	39.69 ± 8.97	42.43 ± 10.43	41.98 ± 10.25
Triglyceride (mg/dL)	185.19 ± 101.01	140.42 ± 82.24	147.82 ± 87.22
Low-density lipoprotein (mg/dL)	126.33 ± 31.87	114.91 ± 30.88	116.80 ± 31.33
Creatinine (mg/dL)	1.06 ± 0.17	1.03 ± 0.16	1.04 ± 0.164
Cholesterol (mg/dL)	200.64 ± 39.51	185.72 ± 37.90	188.18 ± 38.56

*CVD* cardiovascular disease, *BMI* body mass index

Optimal values of hyper-parameters for each algorithm have been reported in Table 2. Based on SHAP values, for the XGBoost model, the most important variables in predicting diabetes were fasting plasma glucose, two-hour postprandial plasma glucose, BMI, waist-hip ratio, age and family history of diabetes with mean absolute values of 0.637, 0.586, 0.356, 0.214, 0.201, and 0.201, respectively. For random forest, top variables were two-hour postprandial plasma glucose, fasting plasma glucose, BMI, age, and triglyceride with mean absolute values of 0.0848, 0.0775, 0.0286, 0.0144 and 0.0125, respectively.

**Table 2:** Optimal hyper-parameters values based on fivefold stratified cross-validation grid search

Model	Hyper-parameters
DNN	Number of layers = 4, number of nodes in each layer = (100,75,50,1), dropout rate in each layer = (0.5,0.5,0.25), activation function in each layer = (ReLU, ReLU, ReLU, sigmoid)
XGBoost	Learning rate = 0.3, maximum depth of each tree = 3, minimum loss reduction to split each node = 1, regularization term on weights = 20, subsample ratio of columns for each tree = 0.5
Random forest	Number of trees in the forest = 1500, maximum depth of each tree = 19, the minimum number of samples to split each node = 8

The results indicated that XGBoost and DNN (except for accuracy) in terms of all metrics outperform random forest (Table 3). In comparison with XGBoost, DNN has higher values in f1-measure, g-mean, and AUROC. Based on MCC, these two algorithms have approximately similar performance, but in terms of AUPRC, XGBoost performs better than DNN.

**Table 3:** Comparison between deep neural network, extremely gradient boosting and random forest based on various metrics in test dataset

	Accuracy	F1-measure	G-mean	MCC*	AUROC	AUPRC	Confusion matrix**	
DNN	0.862	0.575	0.713	0.747	0.857	0.603	0.926	0.074
							0.452	0.548
XGBoost	0.872	0.554	0.667	0.748	0.854	0.622	0.956	0.044
							0.534	0.466
Random forest	0.869	0.543	0.659	0.741	0.840	0.578	0.955	0.045
							0.545	0.455

*MCC* Matthews Correlation Coefficient; *AUROC*Receiver Operating Characteristic Area Under Curve; *AUPRC* Precision-Recall Area Under Curve

^*^MCC has been projected from [-1,1] to [0,1] by $\frac{MCC+1}{2}$ formula

^**^Predicted and actual, non-diabetic and diabetic percent are presented in confusion matrix

**Fig. 4:**
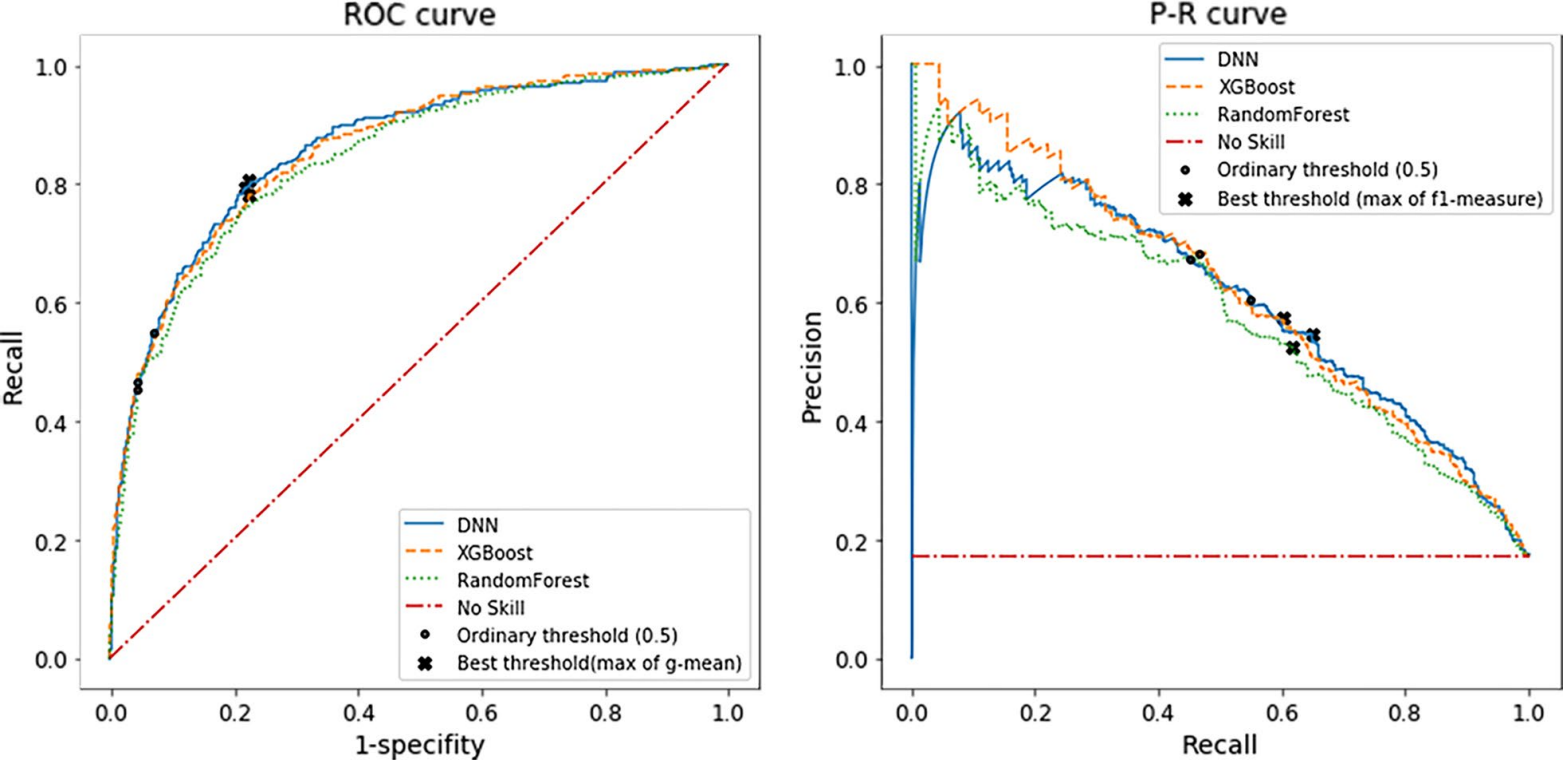
ROC and Precision-Recall curves to find best threshold based on maximum of g-mean and f1-measure for all algorithms. Note: Star marker corresponds to threshold which maximize g-mean in ROC curve and f1-measure in P-R curve

Figure 4 depicts best thresholds that lead to maximum of g-mean and f1-measure in ROC and P-R curves in all algorithms. Based on g-mean criteria, the thresholds are 0.266, 0.12, and 0.168 for DNN, XGBoost and random forest, respectively. For f1-measure these thresholds are 0.427, 0.310, and 0.294 respectively.

Changing threshold from an ordinary value (0.5) to one based on maximum of g-mean has led to higher g-mean, but other metrics have experienced a drop in all algorithms (Table 4). While changing threshold based on maximum of f1-measure yields better performance in f1-measure, g-mean in all algorithms, and MCC in DNN, and XGBoost. The percent of improvements based on f1-measure and g-mean were 1.6, and 4.4 in DNN, 3.2, and 7.1 in XGBoost, and 2.1, and 7.4 in random forest, respectively. MCC has enhanced by 0.3 and 0.1 percent in DNN and XGBoost. Only for random forest, there was a 0.7 percent decrease in MCC. Moving threshold does not affect ROC and P-R AUCs, because they are independent of the selected threshold. Based on both approaches accuracy has decreased in all algorithms.

**Table 4:** Evaluation the effect of moving threshold and weighing in performance of the algorithms

	Accuracy	F1-measure	G-mean	MCC	AUROCC	AUPRC
**DNN**						
g-t	0.784	0.554	0.786	0.732	0.857	0.603
f1-t	0.848	0.591	0.757	0.750	0.857	0.603
weighted	0.822	0.581	0.780	0.744	0.858	0.606
**XGBoost**						
g-t	0.774	0.538	0.774	0.721	0.854	0.622
f1-t	0.855	0.586	0.738	0.749	0.854	0.622
weighted	0.832	0.588	0.776	0.748	0.853	0.620
**Random forest**						
g-t	0.777	0.534	0.767	0.717	0.840	0.578
f1-t	0.841	0.564	0.733	0.734	0.840	0.578
weighted	0.810	0.566	0.775	0.735	0.846	0.591

*g-t* maximum g-mean based moved threshold, *f1-t* maximum f1-measure based moved threshold

Weighing diabetic class in all algorithms in comparison with original models have increased f1-measure, g-mean, AUROC, and AUPRC. Only for weighted XGBoost, there is a slight drop in the ROC and P-R AUCs (0.1 and 0.2 percent, respectively). Among improved metrics, g-mean experienced the most increase by 6.7 percent in DNN, 10.9 percent in XGBoost and 11.6 percent in random forest. When compared to changing threshold based on maximum of g-mean, weighing has boosted performance in terms of accuracy, f1-measure, and MCC in all algorithms and g-mean in XGBoost, and random forest. On the other hand, only g-mean improved in weighted algorithms in comparison with changing threshold based on maximum of f1-measure.

**Fig. 5:**
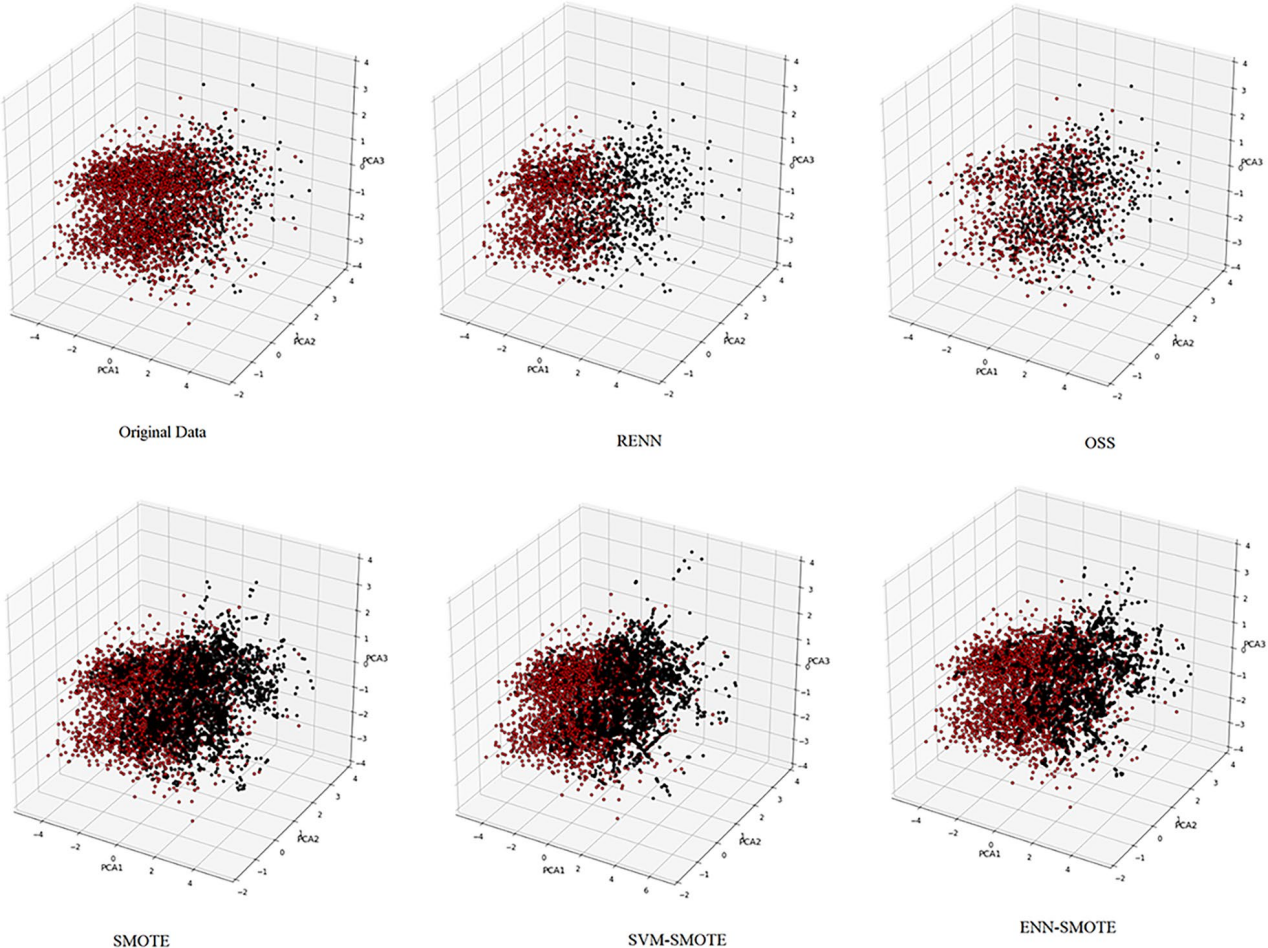
Comparison between various sampling methods on distribution of diabetic (black circles) and non-diabetic (red circles). X, y and z axes are first to third principal components

For the last approach to enhance accuracy in prediction diabetes, we have used 5 sampling methods which their effect on the distribution of classes have shown in Fig. 5.

RENN under-sampling method consistently has increased f1-measure, g-mean, and AUROC in all algorithms (Table 5). Also, AUPRC improved by RENN in DNN algorithm. The other under-sampling method, OSS, only has boosted g-mean in all algorithms. In terms of f1-measure, g-mean, MCC, AUROC and AUPRC, one of under-sampling methods outperforms other sampling strategies in DNN and random forest. For XGBoost algorithm, this superority is based on f1-measure and g-mean metrics.

**Table 5:** Comparison between various sampling methods on the performance of algorithms

	Accuracy	F1-measure	G-mean	MCC	AUROC	AUPRC
**DNN**						
RENN	0.830	0.583	0.773	0.745	0.862	0.608
OSS	0.856	0.594	0.747	0.753	0.855	0.599
SMOTE	0.805	0.556	0.768	0.729	0.855	0.594
SVM-SMOTE	0.827	0.580	0.773	0.743	0.856	0.602
ENN-SMOTE	0.818	0.563	0.763	0.733	0.850	0.599
**XGBoost**						
RENN	0.814	0.572	0.779	0.740	0.856	0.588
OSS	0.831	0.554	0.733	0.727	0.842	0.591
SMOTE	0.859	0.568	0.708	0.742	0.848	0.592
SVM-SMOTE	0.844	0.555	0.718	0.730	0.858	0.605
ENN-SMOTE	0.857	0.548	0.688	0.733	0.845	0.594
**Random forest**						
RENN	0.808	0.556	0.764	0.728	0.844	0.553
OSS	0.832	0.561	0.741	0.731	0.837	0.569
SMOTE	0.842	0.543	0.704	0.724	0.840	0.550
SVM-SMOTE	0.838	0.548	0.717	0.726	0.842	0.552
ENN-SMOTE	0.844	0.531	0.687	0.719	0.843	0.541

In comparison with original data, g-mean in all algorithms, and f1-measure in XGBoost has increaed by SMOTE. While, SVM-SMOTE has resulted in improvement in both g-mean and f1-measure in three algorithms, and AUROC in tree based algorithms. Lastly, AUROC in random forest and g-mean in three classifers have boosted by ENN-SMOTE sampler.

In comparison among sampling methods, based on AUROC, RENN is the best sampling methods in all algorithms and based on AUPRC, SVM-SMOTE in XGBoost, OSS in random forest and RENN in DNN have the best performance.

To summarize the results, for DNN algorithm, best strategies to deal with imbalance issue among three applied approaches are, OSS in terms of accuracy, f1-measure and MCC, and RENN under-sampling methods in terms of ROC and P-R AUCs. For XGBoost algorithm, with approximately same values of MCC, AUROC and AUPRC, weighing yields an improvement of 3.4 and 10.9 percent in f1-measure and g-mean, respectively. In terms of mentioned metrics, weighing, and in terms of AUROC, RENN are the best approaches. For random forest algorithm, as XGBoost, weighing has increased f1-measure and g-mean by 2.3 and 11.6 percent, respectively. In addition to mentioned metrics, AUROC and AUPRC experienced an improvement of 0.6 and 1.3 percent, respectively. Based on all these metrics, as well as MCC, weighing is the best solution to tackle imbalance issue for random forest.

## Discussion

We studied three powerful machine learning algorithms to predict diabetes incidence in the future based on some demographic, biochemical, and anthropometric measures. To tackle minority diabetes class imbalance, we used three strategies. Changing threshold as a simple strategy, cost-sensitive learning and sampling which involve more searching to fit optimal algorithm, are applied.

We evaluated the performance of algorithms before and after providing a solution to the imbalance issue by examining various metrics. Each metric focuses on a special aspect of performance. Except ROC and P-R AUCs, all metrics are constructed based on confusion matrix. Accuracy is consistently decreased after applying imbalance solutions, while g-mean as unbiased metric in imbalanced data [29] is raised substantially. Other metrics had variable behavior.

Our results show that changing threshold based on value that maximizes f1-measure, improved f1-measure, g-mean, and MCC (except for random forest) in three investigated algorithms. In changing threshold approach, the algorithm is not refitted. As a consequence, training time is reduced in comparison with other strategies which imply new hyper-parameters. This effortless solution could have comparable results with other solutions [34]. Our study also demonstrates its efficiency. Although, ROC and P-R AUCs remain constant, for a powerful trained algorithm changing threshold could be a first solution to enhance overall performance and to increase prediction accuracy in minority diabetes class.

For tree-based algorithms, XGBoost and random forest, cost-sensitive learning was the best approach based on f1-measure and g-mean. Besides, it had good results in DNN. In comparison with sampling strategies, weighing only has one hyper-parameter which should be tuned. As a result, the complexity of the training procedure and run-time are lower than sampling methods. By increasing the weight of minority diabetes class, sensitivity is consistently increased but on the other hand, specificity is decreased [39, 40].

Usually, to address the imbalance problem, sampling strategies are applied [41–43]. We studied five sampling methods. Among sampling strategies, one of the under-sampling methods outperformed over-sampling and hybrid procedures based on f1-measure and g-mean in all algorithms. Although in comparison with original data, sampling resulted in better performance, they were not the best solution to solve imbalance distribution between diabetic and healthy classes. Only for DNN, sampling method outperformed other approaches. Sampling strategies have multiple hyper-parameters that should be tuned precisely.

Overall, in original imbalanced data, DNN had highest accuracy for minority diabetes class and outperformed other classifiers based on mean of metrics. After giving solution to class imbalance, in terms of AUROC and AUPRC, under-sampled DNN and weighted XGBoost were better performers, respectively, among combination of algorithms and solving imbalance problem approaches. One of the applied advantages of XGBoost is its ability to model data with missing values which is a common case in medical data [27]. In addition, it is trained very fast and as a powerful algorithm, it has attracted attention in modeling challenging data [44, 45].

One limitation of our work is the low number of investigated sampling methods. SMOTE oversampling is frequently applied to handle class imbalance [12], but in our study, it was not the best performer. A possible explanation for this could be the high overlap between two classes in our data. Applying SMOTE could result in more ambiguous borderline between diabetes and non-diabetes classes. To explore the efficiency of sampling strategies, we will study a larger number of methods in the future with other datasets.

## Conclusion

To conclude, we studied three main approaches to address the class imbalance in predicting diabetes risk. Our optimized algorithms led to a considerable rise in accurate prediction of rare diabetes class before and after giving imbalance solutions for TLGS data [43]. Weighing and changing threshold, compared to resampling methods are faster solutions to handle class imbalance. Our study results could assist researchers to choose the best way to deal with class imbalance for medical data.

## Data Availability

The datasets generated and/or analysed during the current study are not publicly available because this data are only available for approved proposals at Research Institute for Endocrine Sciences (RIES) in Shahid Beheshti University of Medical Sciences but are available from Davood Khalili, head of Department of Biostatistics and Epidemiology at RIES (email: dkhalili@endocrine.ac.ir) on reasonable request.

## Abbreviations

AUC: Area Under Curve
BMI: Body mass index
CART: Classification and regression trees
CVD: Cardiovascular disease
DM: Diabetes mellitus
DNN: Deep neural network
IDF: International Diabetes Federation
MCC: Matthews Correlation Coefficient
OSS: One sided selection
P-R: Precision-Recall
RENN: Repeated edited nearest neighbors
RF: Random forest
ROC: Receiver Operating Characteristic
SMOTE: Synthetic minority oversampling technique
SVM: Support vector machine
TLGS: Tehran Lipid and Glucose Study
XGBoost: Extremely gradient boosting
